# Ion channels and transporters regulate nutrient absorption in health and disease

**DOI:** 10.1111/jcmm.17853

**Published:** 2023-08-28

**Authors:** Xianmin Lu, Chen Luo, Jiangbo Wu, Ya Deng, Xingyi Mu, Ting Zhang, Xiaoxu Yang, Qi Liu, Zhuo Li, Siqi Tang, Yanxia Hu, Qian Du, Jingyu Xu, Rui Xie

**Affiliations:** ^1^ Department of Gastroenterology Digestive Disease Hospital, Affiliated Hospital of Zunyi Medical University Zunyi China; ^2^ The Collaborative InnovAffiliated Hospital of Zunyi Medical Universityation Center of Tissue Damage Repair and Regeneration Medicine of Zunyi Medical University Zunyi China

**Keywords:** disease, ion channels, nutrient, prevention and cure, transporters

## Abstract

Ion channels and transporters are ubiquitously expressed on cell membrane, which involve in a plethora of physiological process such as contraction, neurotransmission, secretion and so on. Ion channels and transporters is of great importance to maintaining membrane potential homeostasis, which is essential to absorption of nutrients in gastrointestinal tract. Most of nutrients are electrogenic and require ion channels and transporters to absorb. This review summarizes the latest research on the role of ion channels and transporters in regulating nutrient uptake such as K+ channels, Ca2+ channels and ion exchangers. Revealing the mechanism of ion channels and transporters associated with nutrient uptake will be helpful to provide new methods to diagnosis and find potential targets for diseases like diabetes, inflammatory bowel diseases, etc. Even though some of study still remain ambiguous and in early stage, we believe that ion channels and transporters will be novel therapeutic targets in the future.

## 1 INTRODUCTION

The basic physiological function of the gastrointestinal (GI) tract is to digest and absorb nutrients from the diet and to excrete unabsorbed or harmful substances. Meals are digested enzymatically into absorbable nutrients such as monosaccharides, dipeptides, free amino acids and fatty acids. Absorption of the above nutrients requires specific transporters accompanied by cotransport or reverse transport of ions (Na^+^, H^+^, Cl^−^ or HCO_3_^−^). Establishing and maintaining the driving forces (membrane potential and transmembrane ion gradients) is essential for maximum uptake. Firstly, it was found that changes in membrane potential (Em) affecting nutrient absorption. Membrane potential is primarily controlled by voltage‐gated K+ channel (Kv) activity through electrophysiological experiments, fluorescence detection, RT‐PCR, QPCR and immunohistochemistry. Reducing Kv channel activity by applying Kv channel inhibitors leads to membrane depolarization. Membrane depolarization or hyperpolarization can modulate the activity of various nutrient transporters on intestinal epithelial cell (IEC).^1^, ^2^ Secondly, Simpson JE, Walker NM, Supuran CT et al. found that changes in the cellular ion chemical gradient or pH gradient can also affect nutrient uptake constructing a mouse model for short‐circuit current measurement. The peptide transporter1 (PEPT1) uses a proton electrochemical gradient as the driving force, and its acid‐loading activity requires an apical Na^+^/H^+^ exchangers or an anion exchangers to reduce epithelial acidification.^3^, ^4^ In addition, as an important intracellular secondary messenger, researchers have confirmed that Ca^2+^ regulates many intracellular physiological activities and plays an important role in regulating intestinal nutrient absorption through perfusion techniques in the small intestine of rabbits and mice.^5^, ^6^, ^7^ Intracellular Ca^2+^ ([Ca^2+^]_i_) homeostasis is finely controlled by Ca^2+^ channels and transporters which may ultimately affect nutrient absorption (Table 1).

**TABLE 1:** Modulators of nutrient absorption related Ion channel and transporters and their therapeutic value.

Channels/Transporters	Subtype	Activators	Inhibitors	Therapeutic values	Reference
K^+^	Kv1.1		TEA	Kv1.1 and Kv1.3 channels are involved in the regulation of intestinal glucose absorption.	^35^
	K_v_1.3		PAP‐1		
	K_v_7.1		HMR 1556	Kv7.1 is required for membrane repolarization during electrogenic transport.	^33^
	K_Ca_3.1		Clotrimazole	KCa3.1 (KCNN4) plays a major role in regulating H^+^/dipeptide absorption	^35^
	Kca		NS6180	K^+^ channel is involved in te repolarization of cell membranes, which is essential for stabilizing the driving force of electrogenic nutrient transport.	31, 32
	Kir		Polyamine	Kir involves in the regulation of the resting membrane potential of cells.	
	K2p		Carvedilol	K2p stabilizes the resting membrane potential and promote the action potential repolarization.	
Ca^2+^	Cav1.3		Nisoldipine	Cav1.3 Regulates many intracellular physiological activities and intestinal nutrient absorption.	^9^
SOCE			2‐APB	Ca^2+^channel (SOC) mediates the influx of calcium ions during storage operations.	^39^
PKC‐α			Aprinocarsen	Leukotriene D4 (LTD4) promotes the phosphorylation of ASCT1 through the PKC‐α pathway, and reduces affinity to inhibit Na^+^‐alanine cotransport in IEC during chronic intestinal inflammation.	^95^
SGLT1			Phlorizin	SGLT1 is the secondary active transporter that translocates one d‐glucose molecule together with two sodium ions into cells employing the inwardly directed sodium gradient that is generated by the (Na/K)‐ATPase as driving force.	^13^
GLUT2			Cytochalasin B	GLUT2 Transports d‐glucose, d‐neneneba galactose and fructose in human body.	^13^
PEPT1			Floxuridine	PEPT1 can achieve proton coupled absorption of over 8000 different dipeptides and tripeptides.	^13^
FATP4			T0070907	FATP4 actively transports medium‐chain and long‐chain fatty acids (MCFA and LCFA) across the apical membrane of intestinal cells.	^17^
MCT1 SMCT1			Ibuprofen	MCT1 and SMCT1 mediated active transport or exchange with bicarbonate (HCO3^−^).	22, 23, 24
NPC1L1			Bexarotene	NPC1L1 can promote cholesterol uptake by facilitating the passage of sterols through the brush‐like border membrane (BBM) of intestinal cells.	^18^
NCX	NCX1		TGF‐β	NCX1 promotes the secretion of bicarbonate (HCO_3_^−^) in the duodenum, and promotes the absorption of glucose and glutamine.	72, 73
ABCG5/8			GW3965	ABCG5/8 promotes the secretion of active cholesterol by intestinal cells.	^18^
NBC	NBCe1		Acetazolamide	NBC mediates intestinal uptake of HCO_3_.	^88^
NHE	NHE3		S1611	Apical NHE3 is essential for the intestinal absorption of various nutrients and minerals such as Ca^2+^, glucose, fatty acids and amino acids	78, 79, 88
TRPV	TRPV1	Capsaicin	Ruthenium red	TRPV1 activation will mediates the secretion behaviour of intestinal endocrine cells in a Ca^2+^‐dependent manner	^53^
	TRPV4	Tetrahydrocannabivarin	Capsaicin	Capsaicin could promote Na^+^‐glucose absorption by inhibiting TRPV4 channel.	^55^
VGCC			Nifedipine	Nifedipine as an L‐type VGCC blocker could attenuate the effect of glucose.	^66^

Nutritional disorders are more or less present in many diseases, which may exacerbate the condition and affect the progress of recovery. Given that enteral nutrition (EN) remains the preferred option, findings from epidemiology study, animal study and clinical study have proved that the treatments targeted at IEC malabsorption may be more effective and safer.^8^, ^9^, ^10^ Most recent studies of controlling nutrient absorption by ion transport in the gut have focused on neural and hormonal regulation^11^, ^12^; there are few studies on regulating nutrient absorption by IEC ion channels and transporters. This review does not address the role of intestinal neuroendocrine and paracrine factors on nutrient absorption; we focus on the functional and molecular mechanisms of IEC ion channels and transporters involved in the regulation of nutrient absorption in physiological and pathophysiological states, particularly in diabetes and inflammatory bowel disease (IBD).

## 2 MECHANISMS OF NUTRIENTS ABSORPTION IN THE INTESTINE

### 2.1 Absorption of sugars in the intestine

Dietary carbohydrates must be broken down into monosaccharides (such as glucose and galactose) before they can be transported and absorbed. Intestinal monosaccharide uptake mainly depends on sodium‐glucose cotransporter 1 (SGLT1) and glucose transporter 2 (GLUT2). SGLT1 and GLUT2 were detected primarily in the small intestine (duodenum, jejunum and ileum) but not in the oesophagus, stomach, colon or rectum.^13^ Take glucose absorption, for example; low‐concentration glucose (< 30 mM) in the lumen is cotransported with 2 Na^+^ to epithelial cells through SGLT1 of brush border membrane (BBM). GLUT2 of the basolateral membrane (BLM) transports glucose from IEC to mesenteric veins. In addition to the classical pathways described above, active glucose transport saturates in the presence of high concentrations of glucose (> 30 mM) in the intestinal lumen, possibly involving intercellular glucose transport and GLUT2 can rapidly bind to the brush border membrane of enterocytes and participate in promoting glucose diffusion across the membrane.

### 2.2 Absorption of proteins in the intestine

Dietary proteins are cleaved into oligopeptides in the lumen and further processed into small peptides (dipeptides and tripeptides) and free amino acids (FAA). Ma GG et al. constructed a mouse model, and conducted haematoxylin and eosin (HE) staining, enzyme‐linked immunosorbent assay (ELISA) and other experimental studies to show that PEPT1 (SLC15A1), a high‐capacity/low‐affinity peptide transporter, can achieve proton coupling absorption of more than 8000 different dipeptides and tripeptides, a variety of peptide‐like compounds are also PEPT1 transport substrates, including specific immune stimulants, angiotensin‐converting enzyme inhibitors and β‐lactam antibiotics.^14^, ^15^ The peptides in IECs are further hydrolyzed to FAA, and a small part of the absorbed oligopeptides are transported out by the H^+^‐dependent peptide transport system on the basement membrane. At the same time, FAA is mediated by several specific amino acid transporters. Most of the FAA in the lumen are absorbed by Na^+^‐dependent cotransporters (such as system A, ASC, B^0^), while a small portion of the FAA enters intestinal cells with other ions such as H^+^, protein associated with topoisomerase (PAT), Cl^−^ or other amino acids.^16^ IECs could convert or metabolize a portion of amino acids (e.g. glutamine, glutamate and aspartate) for their own use. The other part is transported out through the transport system of the basolateral membrane into the portal circulation.

### 2.3 Absorption of lipids in the intestine

Hydrolysis products of dietary lipids, such as fatty acids, glycerol esters and cholesterol, do not dissolve in water. They form water‐soluble micelles with bile salts before reaching the microvilli through the hydrostatic layer on the mucosal surface of the small intestine. Then fatty acids, glycerol esters, etc., are released from micelles and enter the intestinal epithelium through the membrane, while bile salts return to the lumen. In vivo and in vitro studies on weaned piglets and the IPEC‐J2 cell line indicate that fatty acid translocase (CD36) and fatty acid transporter 4 (FATP4) actively transport medium‐chain and long‐chain fatty acids (MCFA and LCFA) across the apical membrane of intestinal cells.^17^ The Niemann–Pick C1‐like intracellular cholesterol transporter Niemann–Pick C1‐like 1 protein (NPC1L1) is a sterol influx transporter located in the apical membrane of intestinal cells. It can actively promote cholesterol uptake by facilitating the passage of sterols through the brush‐like border membrane (BBM) of intestinal cells. ATP‐binding cassette transporters (ABCG5/8) promote active cholesterol excretion from intestinal cells.^18^, ^19^ These intracellular lipids (medium‐chain fatty acid (MCFA), long‐chain fatty acid (LCFA) and cholesterol) participate in the formation of chylomicrons (CM) in the presence of apolipoprotein‐B48 (apoB48) and microsomal triglyceride transfer protein (MTTP), which cross the basolateral membrane and enter the lymph.^20^, ^21^ The in vitro experiments including enzyme‐linked immunosorbent assay, immunoblotting analysis proved that the production of short‐chain fatty acids (SCFAs) by the fermentation of dietary fibre by commingled enterobacteria in the colon is mainly through monocarboxylate transporter 1 (MCT1) and sodium‐coupled monocarboxylate transporter 1 (SMCT1) mediated active transport or exchange with bicarbonate (HCO_3_^−^). The colon takes a fraction of undissociated SCFAs through passive diffusion into the mesenteric venous blood and then the portal vein.^22^, ^23^, ^24^

## 3 METHODS FOR STUDYING INTESTINAL NUTRIENT ABSORPTION

Various animals and their isolated intestinal segments, brush border membrane vesicles, cultured organoids or cell lines are used in vivo and in vitro to study nutrient absorption. The material balance test is an intuitive method to measure the absorption of nutrients in the intestine. The concept of the ‘ingestion minus output’ technique measures the amount remaining at a point in the digestive tract, for example, in ileostomy output or stool. Although the concept is simple, the practice in vivo is difficult because of the need to measure dietary composition and consumption accurately. It also requires consideration that bacterial consumption is also involved in the loss of nutrients after ingestion. Radioisotope labelling is used to study the absorption of various nutrient‐like substances in vivo and in vitro experiments better than mass balance measurement, usually with [^3^H] or [^14^C] isotopes.^25^ Dietary triacylglycerol absorption was measured by simultaneous feeding of [^133^I] ‐triolein and [^75^Se] ‐glyceryl triether, and cholesterol absorption was measured with radiolabeled dietary plant sterols (e.g. sitosterol or sitosterol) as a nonabsorbable reference marker.^26^ Some nonradioactive markers reflect the absorption of specific nutrients. For instance, D‐alanine‐lysine‐N‐7‐amino‐4‐methylcoumarin‐3‐acetic acid (D‐Ala‐Lys‐AMCA) is a fluorophore conjugative peptide molecule, which is used as a reporter molecule for peptide transport.^27^ A polyester made by esterification of sucrose and behenic acid (‘sucrose polybehenate,’ SPB) as a marker to evaluate intestinal fatty acid absorption.^28^ Ussing chamber is commonly used to study the transport of epithelial nutrients and electrolytes in vitro. The short‐circuit current (*I*_*sc*_) or mucosal‐to‐serosal fluxes (*J*_*ms*_) induced by the addition of nutrients to the apical chamber of tissues, or epithelial cells would represent the process of nutrient absorption.^29^ Using the electrophysiological measurements requires nutrient uptake accompanied by ion transmembrane transport, such as amino acids and glucose uptake accompanied by Na^+^ and dipeptide transport accompanied by H^+^.

## 4 INTESTINAL CHANNELS MEDIATE NUTRIENT TRANSPORT

### 4.1 The K^+^‐channels in the intestinal nutrient uptake

**FIGURE 1:**
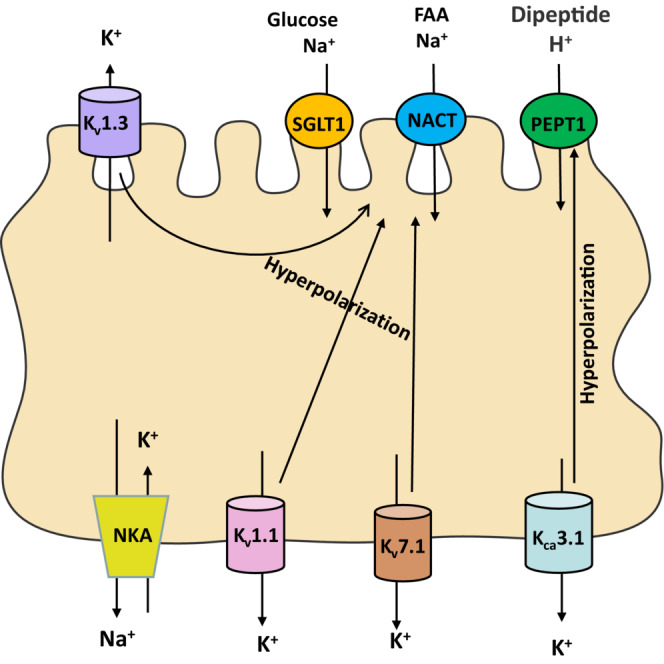
The K^+^‐channels in the intestinal nutrient uptake.

K^+^ transmembrane transport via K^+^ pumps and channels occurs in mammalian excitable or inexcitable cells. These transports involve various physiological functions, such as hormone secretion and rhythm regulation. K^+^ channels are the most isoform and the most complex ion channels, which can be divided into the following categories: calcium‐activated (K_Ca_), inwardly rectifying (K_ir_), voltage‐gated (K_v_) and two‐pore (K_2p_) K^+^ channels. K^+^ channels are involved in the repolarization of cell membranes, which is essential for stabilizing the driving force of electrogenic nutrient transport. Several subtypes of K^+^ channels have been described in IEC, including K_v_1.1 (KCNA1), K_v_1.3 (KCNA3), K_Ca_3.1 (KCNN4) and K_v_7.1 (KCNQ1) (Figure 1), and have been proved to regulate ion secretion, migration of epithelial cells and other functions.^30^, ^31^, ^32^

Volker Vallon et al. used Ussing Chamber to study nutrient transport in mouse jejunum. *I*_*sc*_ generated by glucose and phenylalanine represent absorption processes, and jejunal currents were significantly attenuated after gene knockout or drug inhibition of K_v_7.1 (KCNQ1), suggesting that basolateral K_v_7.1 is required for membrane repolarization during electrogenic transport.^33^ Ailin Xiao et al. showed similar results in the small intestine of rats verifying the expression and location of Kv subtypes by RT‐PCR, Western blotting and immunohistochemistry to suggest that basolateral K^+^ channels maintain glucose and amino acid absorption but did not identify specific K^+^ channels.^34^ Chao Du et al. studied the role of K_v_ channel subtypes in the mouse jejunum, demonstrating that K_v_1.1 and K_v_1.3 channels are functionally expressed on the serosal and mucosal sides, respectively, through in vivo experiments, such as immunofluorescence, PCR and protein imprinting.^35^ They regulate intestinal glucose absorption, and inhibitors of these channels attenuate glucose transport. In addition to Na^+^ coupled nutrient transport, recent studies have found that the basolateral K_Ca_3.1 (KCNN4) plays a major role in regulating H^+^/dipeptide absorption. K^+^ induces membrane hyperpolarization, thereby increasing the driving force of H^+^‐coupled electrogenic dipeptide absorption. Currently, there is no direct evidence that K^+^ channels in IEC regulate lipids absorption; it is still worth studying.

### 4.2 The Ca^2+^‐channels in the intestinal nutrient uptake

**FIGURE 2:**
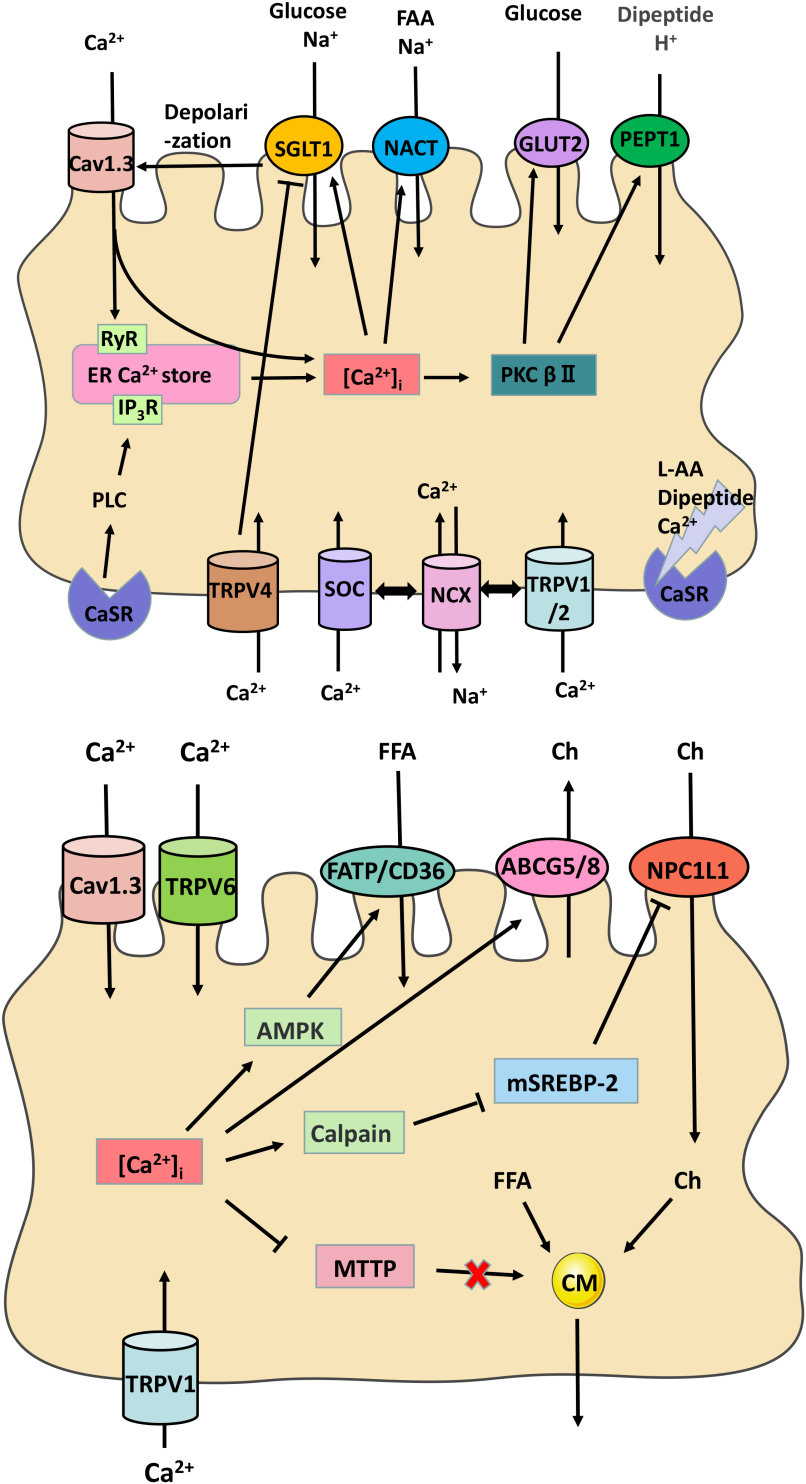
The Ca^2+^‐channels in the intestinal nutrient uptake.

Ca^2+^ is an important substance involved in various life activities, such as muscle contraction, the release of hormones and neurotransmitters, blood coagulation, etc., and the absorption of Ca^2+^ from the lumen is the main supply. It is generally believed that there are two uptake pathways of intestinal Ca^2+^: transcellular and paracellular. (Figure 2). The transcellular pathway mainly occurs in the proximal intestine through the apical Ca^2+^ channels (TRPV5/6 primarily exists in the duodenum, and Cav1.3 is most abundant in the distal jejunum and proximal ileum).^36^ Intracellular Ca^2+^ ([Ca^2+^]_i_) transport is mainly mediated by calcium‐binding proteins, including calbindin‐d9k and calmodulin (CaM), which may play a role in the buffering system to prevent [Ca^2+^]_i_ overload and transfer Ca^2+^ to the serosal side. The [Ca^2+^]_i_ are actively excreted by plasma membrane Ca^2+^‐ATPase (PMCA) and other transporters such as Na^+^/Ca^2+^ exchanger (NCX).^37^ In addition to the above classical pathways, more serosal channels in IEC were discovered to mediate Ca^2+^ influx, such as TRPV1/4 and store‐operated Ca^2+^ channels (SOC).^38^, ^39^, ^40^ Extracellular calcium‐sensitive receptor (CaSR) is one of the important nutrient‐sensing receptors.^41^ Activation of CaSR mediates [Ca^2+^]_i_ homeostasis through the GPCR‐PLC‐IP_3_R and GPCR‐PLC‐TRPV6 pathway.^42^, ^43^, ^44^ Stable [Ca^2+^]_i_ signalling through the above pathways regulates intestinal physiological functions, including barrier protection, enteral hormone secretion and motility. And nutrient transport is usually accompanied by [Ca^2+^]_i_ movement, [Ca^2+^]_i_ signalling more or less mediated the nutrient's absorption.^45^, ^46^, ^47^

Hyson, D H et al. applied calcium channel blockers (nisoldipine) in rabbit jejunum. They found that D‐glucose uptake was decreased, suggesting that calcium channel blockers could affect the active transport of glucose through SGLT1 to different degrees.^48^ Emma L. Morgan et al. studied that SGLT1 transport of glucose can induce rapid insertion and activation of GLUT2 in the apical membrane through a Ca^2+^‐PKCβII dependent mechanism. The Ca^2+^ influx in this process mainly originates from the Cav1.3 activated by depolarization.^49^, ^50^ According to a recent study by Fenglian Zhang et al., when glucose uptake leads to the activation of Cav1.3 channels, the Ca^2+^ influx through Cav1.3 activates the ryanodine receptor (RyR1) to release the endoplasmic reticulum (ER) Ca^2+^ storage, thereby inducing store‐operated Ca^2+^ entry (SOCE) mechanism. The increased [Ca^2+^]_i_ caused by the above transports further activates SGLT1 to mediate intestinal glucose uptake.^51^ Andras Domotor et al. studied that capsaicin increases glucose uptake and utilization in a TRPV1‐dependent manner in healthy humans. The main consideration is the effect of intestinal hormones; Transient receptor potential vanilloid 1(TRPV1) activation stimulates the secretion of intestinal endocrine cells in a Ca^2+^‐dependent manner.^52^ When TRPV1 as nonselective cation channels are activated by their well known agonist capsaicin, sodium and calcium ions would enter through TRPV1 channels to depolarize nociceptive neurons, leading to action potential firing and finally the sensation of spiciness.^53^ However, Wan et al. recently found that capsaicin enhanced glucose absorption in the jejunum of wild‐type (WT) mice. Applying a TRPV1 blocker did not alter this response, but the TRPV4 agonist did. In addition, TRPV4 KO mice absorbed glucose more strongly than WT mice. Therefore, it is suggested that capsaicin can promote Na^+^‐glucose absorption by inhibiting TRPV4 channel.^54^

Studies in Caco‐2 cells showed that reducing Ca^2+^ levels stimulated Na(+)/H(+) exchanger 3 (NHE3) activity, thereby maintaining the high electrochemical driving force of PEPT1 to drive peptide transport.^55^ Similarly, Yukiko et al. found that capsaicin interacts with TRPV1 to reduce PEPT1‐mediated transport in rat intestines; the inhibitory effect was attenuated by intravenous administration of ruthenium red, a nonselective inhibitor of TRP channels.^56^ However, a recent study shows that [Ca^2+^]_i_ activated Na^+^‐glutamine absorption in ileal epithelial cells via several Ca^2+^ permeable channels and transporters, including mucosal Cav1.3, serosal TRPV1/2, SOC channels and NCX.^57^ And it is generally accepted that peptides and L‐AA activate Ca^2+^ ‐sensing receptors (CaSR) and serve as allosteric modifiers to nutrient signal supply to intestinal epithelial cells (IEC).^58^, ^59^ Jingyu Xu et al. found that [Ca^2+^]_i_ stimulates the positive feedback pathway of peptide absorption. The absorbed Gly‐Sar stimulates basolateral CaSR, leading to the activation of phospholipase C (PLC) and the increase of [Ca^2+^]_i_, which then activates IK_Ca_ to provide the driving force for PEPT1 transepithelial uptake.^60^

In the study of lipids absorption, early research studied that luminal Ca^2+^ increases the absorption of LCFA in rat jejunum; there is a potential for that Ca^2+^ transport and LCFA transport to be correlated in the brush border and the basolateral membrane of IEC.^61^ Calcium channel blockers (CCBs) affect plasma lipid level and lipoprotein metabolism; Emile Levy et al. found reduced chylomicron output in rats treated with TA‐3090 by gavage, indicating reduced intestinal fat absorption and reduced postprandial hypertriglyceridaemia.^62^ The application of different CCBs (nisoldipine and verapamil) in the jejunum of rabbits by D.A. Hyson et al. showed different effects on various lipid component uptake, which correlated with the dietary proportion of cholesterol (Ch).^63^ Ka Ying Ma et al. investigated that dietary Ca^2+^ reduces plasma cholesterol by downregulating intestinal (NPC1L1) and MTTP, upregulating hepatic cholesterol‐7α‐hydroxylase (CYP7A1) and intestinal ABCG 5/8.^64^ Then, Gulsum E. Muku et al. studied that NPC1L1 expression in the liver and intestine depends on sterol regulatory element binding protein 2 (SREBP‐2) activity, aryl hydrocarbon receptor (AHR) induces proteolytic degradation of mature SREBP‐2 (mSREBP2) in a Ca^2+^‐dependent manner in Caco‐2 cells, which is associated with AHR ligand‐mediated upregulation of membrane Ca^2+^ channels encoded by transient receptor potential cationic channel (TRPV6) gene.^65^

Hiroshi Arakawa et al. found that the nutrient absorption and energy supply network composed of the Cav1.3‐Ca^2+^‐PKCβII mechanism mainly coordinated glucose absorption and PEPT1‐mediated peptide absorption. Glucose decreased the expression of PEPT1 in the jejunal membrane of rats, and nifedipine as an L‐type voltage‐gated calcium channels (VGCC) blocker could attenuate the effect of glucose using perfused rat jejunum in vivo.^66^ We considered another possible Ca^2+^ signalling pathway linking nutrients uptake. [Ca^2+^]_i_ binds CaM as a compound that activates calmodulin‐dependent protein kinase β (CaMKK β). Previous literature has shown that AMP‐activated protein kinase (AMPK) is a substrate for CaMKK2 in mammalian cells from rat brain or expressed in E. coli phosphorylates,^67^ and AMPK is a key sensor of intracellular energy status and affects the expression and transport function of almost all intestinal nutrient transporters (such as SGLT1, GLUT2, PEPT1 and CD36).^68^, ^69^, ^70^, ^71^ In summary, Ca^2+^ signalling could regulate the activities of NHE, K^+^ channels and targeted transporters, which are involved in nutrient transport. It can be seen that the mechanism is complex and still needs to be further studied.

## 5 INTESTINAL ION TRANSPORTERS MEDIATE NUTRIENT TRANSPORT

**FIGURE 3:**
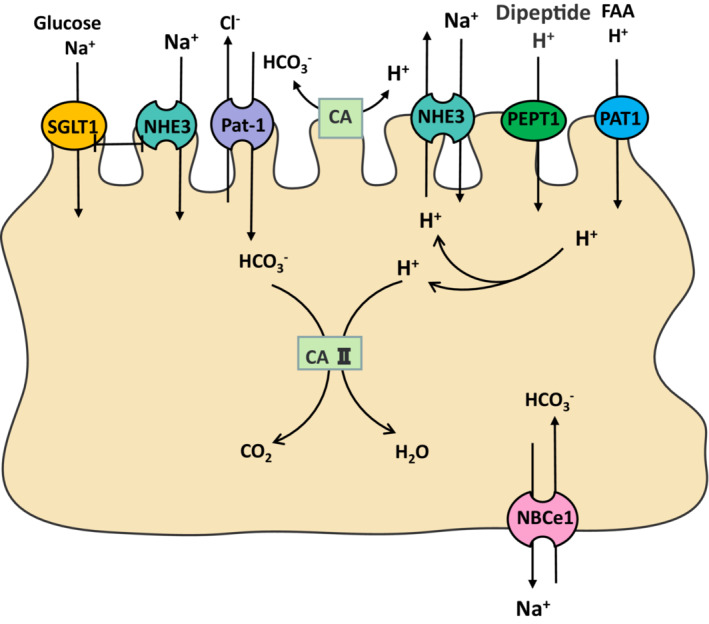
Intestinal ion transporters mediate nutrient transport.

The importance of [Ca^2+^]_i_ and [Na^+^]_i_ in nutrient absorption is recognized; in addition to the above channels, there are also transporters to maintain ion homeostasis. There are currently three subtypes of Na^+^‐Ca^2+^ exchangers (NCX) expressed in various cells, including IEC. The transport ratio of NCX is 3:1; in forward transport mode: 3 Na^+^ are transferred in and 1 Ca^2+^ is moved out of the cell, while in reverse transfer mode is the opposite. The direction depends on the gradient of Na^+^ and Ca^2+^ concentrations and membrane potential (Em). (Figure 3). Dong Hui et al. have shown that NCX1 is expressed in the small intestinal mucosa of mice, and its antitransport mode increases [Ca^2+^]_i_ concentration and promotes duodenal bicarbonate (HCO_3_^−^) secretion.^72^, ^73^ Recent studies have shown that functional and physical interactions between nonselective cation channels (such as TRP) and NCX proteins, mediated Na^+^ and Ca^2+^ influx can affect the direction of NCX transport through blot analysis, immunofluorescence analysis, expression profile and qPCR were conducted in cell experiments.^74^, ^75^, ^76^, ^77^ And then Fenglian Zhang and Fenglan Chu et al. demonstrated that such a mechanism drives the NCX1 antitransport pattern in the small intestinal epithelium to increase [Ca^2+^]_i_ and promote glucose and glutamine absorption.^51^, ^57^

Nutrient uptake is a process of cellular acidification that would reduce the driving force for material transport; maintenance of these absorptions must depend on intracellular buffering or reducing proton loading. Na^+^‐H^+^ exchangers (NHE) are the main mechanism of cell alkalization by mediating H^+^ discharge. We have also applied Ussing chambers to measure net Ca^2+^ absorption across different segments of wild‐type mouse intestine, and found that more than 10 NHE isoforms have been identified in mammals. Apical NHE3 (SLC9A3) is essential for the intestinal absorption of various nutrients and minerals such as Ca^2+^, glucose, fatty acids and amino acids.^78^, ^79^ It is recognized that NHE3 and SGLT1 in the brush border membrane (BBM) of villus cells are the major Na^+^ absorption pathways in the mammalian small intestine. Through uptake and molecular studies to determine NHE3 and SGLT1 activity, Steven Coon et al. showed a significant increase in SGLT1 activity, mRNA and protein in BBM after silencing NHE3 expression in rat intestinal epithelial cells (IEC‐18), suggesting enhanced Na^+^ ‐glucose coabsorption.^80^ The optimal intestinal absorption capacity of certain H^+^‐coupled cotransporters such as PEPT1 and amino acid transporter SLC36A1 (PAT1) depends on the function of the NHE3, which maintains their driving force.^81^, ^82^ In addition to the role of H^+^, Noriko Ishizuka et al. used NHE3 inhibitor and gene‐deficient mice (cldn15 −/−) to disrupt luminal Na^+^ homeostasis, demonstrating that luminal Na^+^ is essential for peptide absorption in native epithelial cells, and there is a new relationship between PEPT1 and NHE3.^83^ C. Schoeller. R et al. found that NHE also affected lipids absorption; inhibiting Na^+^/H^+^ exchange by amiloride reduced oleic acid absorption into the rat jejunal layer. Most fatty acids become protonated (FAH) in the aqueous phase of an acidic microclimate with pH 5.5, which is produced and maintained by H^+^ secreted by the NHE3 and also involves the activation of plasma membrane fatty acid binding protein (FABP‐pm).^84^, ^85^

Cystic fibrosis transmembrane regulator (CFTR), downregulated in adenoma (DRA, SLC26A3), putative anion transporter 1 (Pat‐1, SLC26A6) and anion exchanger 4 (AE4, SLC4A9), which transport Cl^−^/HCO_3_^−^ to control pH_i_. A study by A. K. Stewart et al. in mouse IEC showed that intracellular carbonic anhydrase (CA) activity promotes H^+^ motility, thereby maintaining the transmembrane ion gradient for maximum PEPT1 uptake using intracellular carboxy‐SNARF‐1 fluorescence in combination with whole‐cell microspectrofluorimetry or confocal microscopy.^86^ In the study of expression and function colocalization, Pat‐1 was found to colocalize with PEPT1 in the apical epithelium membrane.^87^ Subsequently, Janet E. Simpson et al. studied that CA provided the source of HCO_3_^−^ for Pat‐1, and intracellular CA could convert HCO_3_^−^ and H^+^, thereby counteracting cell acidification and maintaining PEPT1 transport.^3^ Alternatively, they also suspected that CA might interact with other HCO_3_^−^ transporters, such as the basolateral membrane Na^+^/HCO_3_^−^ cotransporter (NBC), the role of these enzymes may be much more complex. NBC is present basolateral in the whole gastrointestinal epithelium, and intestinal HCO_3_^−^ uptake is assumed to be mediated by such cotransporters. Basolateral NBCe1 (SLC4A4) plays a rate‐limiting role in HCO_3_^−^ secretion. Qin Yu et al. used the Ussing chamber and found that Gly‐Sar‐induced *I*_*sc*_ was reduced in the jejunum of NBCe1 KO mice. NBCe1 is a key steady‐state mechanism that ensures peptide uptake.^88^ From this, the acid load generated by most modes of nutrient absorption in the small intestine could activate NBCe1, which in turn plays a role in maintaining pH and Em during all nutrient absorption.

## 6 ELECTROLYTE TRANSPORT REGULATES INTESTINAL NUTRIENT ABSORPTION IN DISEASE

### 6.1 Diabetes mellitus

Diabetes mellitus is a metabolic disease characterized by hyperglycaemia, which can lead to chronic damage and dysfunction of various tissues, especially eyes, kidneys, nerves and cardiovascular systems. Delaying carbohydrate absorption in the intestine has been an important therapeutic strategy of diabetes, especially in type 2 diabetes mellitus (T2DM) caused by eating disorders. Chao Du et al. studied that basolateral Kv1.1 and apical Kv1.3 inhibitors significantly decreased blood glucose and attenuated weight gain in diabetic mice.^35^ Peijian He et al. concluded that NHE3 activity and fluid absorption in the intestinal tract of streptozotocin‐induced (STZ‐induced) diabetic mice were reduced, restoring Na^+^/H^+^ exchanger NHE3 could improve the fluid loss associated with type 1 diabetes mellitus (T1DM) diarrhoea.^89^ However, Leo K.Y. Chan et al. showed that NHE3 expression is elevated in *db/db* mouse jejunal BBM and high‐glucose‐treated human Caco‐2 cells. NHE3 blockade impaired SGLT1‐mediated intestinal glucose absorption through serum/glucocorticoid regulated kinase 1(SGK1) and improved glucose intolerance in diabetic mice, proposing the NHE3‐SGLT1 signalling axis, potential clinical use of NHE3 blockers in reducing intestinal glucose uptake and counteracting postprandial glucose levels in patients with T2DM.^90^ This difference in the NHE3 expression profile may be related to the differences in the models used, which should be considered when NHE3 drugs are used to treat diabetes.

### 6.2 Cystic fibrosis (CF)

Cystic fibrosis is an autosomal recessive disease caused by mutations in the CF gene on chromosome 7, affecting organs including the ileum, pancreas and lung. The patient had defective chloride channel regulation in epithelial cells and exocrine gland (Galeati's glands, pancreas, sweat glands, etc.) dysfunction. Clinical manifestations included recurrent airway obstruction, infection, intestinal obstruction, dyspepsia, etc. CF has long been considered a malabsorption disorder, especially lipids, partly due to the decreased luminal pH caused by low HCO_3_^−^ secretion leading to decreased pancreatic enzyme efficiency and bile acid precipitation.^91^ CFTR knockdown results in increased intracellular apolipoprotein levels and microsomal transfer protein activity, two important factors for the efficient assembly and secretion of lipoproteins. However, NPC1L1‐mediated cholesterol uptake was unaffected by CFTR gene modification. The role of CFTR in the absorption of carbohydrate and protein nutrients is controversial.^92^ Dipeptide absorption is unchanged in cystic fibrosis patients, whereas neutral amino acid transport is enhanced or decreased in CF patients, and electrical absorption of glucose is increased or unchanged.^93^ But Robert C. De Lisle et al. found that the expression of enterocyte maturation markers involved in nutrient assimilation was greatly reduced in the small intestine of CF mice, including genes/proteins for all macronutrients (carbohydrates, lipids and proteins) and micronutrients.^94^

### 6.3 Inflammatory bowel disease (IBD)

Inflammatory bowel diseases (IBD) include ulcerative colitis (UC) and Crohn's disease (CD), which can cause damage to the whole digestive tract. Its pathogenesis is thought to be a complex combination of the genetic, gut microbiome, immune response and environmental factors, leading to excessive and abnormal immune responses to the symbiotic microbiota. Diarrhoea is a very common debilitating symptom in patients with IBD, and several causes, including malabsorption, can cause it. One reason for disrupting nutrient absorption is that the activity of Na^+^, K^+^ ‐ATPase (NKA), a key driver of nutrient uptake, is reduced in IBD. Na^+^‐dependent neutral amino acid transport systems, such as alanine (Ala) and glutamine (Gln) cotransporters, are significantly affected during IBD, leading to the malabsorption of these essential nutrients. Leukotriene D4 (LTD4), an immune inflammatory mediator, can increase [Ca^2+^]_i_, promote phosphorylation of ASCT1 through the PKC‐α pathway, and reduce affinity to inhibit Na^+^‐alanine cotransport in IEC during chronic intestinal inflammation.^95^ Gln supplements play an important role in the pathogenesis and treatment of IBD.^96^, ^97^ Na^+^‐glutamine cotransporter B^0^AT1 in villus cells was inhibited secondary to reduced cotransporter numbers and reduced NKA activity. SNAT5/SN2 in crypt cells was stimulated secondary to increased transporter affinity and NKA activity.^98^ LTD4 has been shown to stimulate SN2‐mediated glutamine uptake in chronic enteritis. Then Niraj Nepal et al. found that LDT4 elevated [Ca^2+^]_i_ through leukotriene receptors, stimulating NKA in intestinal crypt cells via the PKC pathway.^99^ The TRP channels could affect IBD by regulating visceral sensitivity and submucosal vascular perfusion.^100^, ^101^ Capsaicin has a variety of pharmacological effects (such as anti‐inflammatory, antioxidant and anticancer), and it has recently been shown to enhance gastrointestinal mucosal defence and have therapeutic effects on IBD.^102^, ^103^ Capsaicin is commonly thought to be a TRPV1 agonist; however, the expression of TRPV1 was decreased in the colonic epithelium of UC patients compared with the healthy group, while the TRPV4 was significantly increased.^104^ Wan H et al. administered capsaicin to the dextran sodium sulphate (DSS)‐induced UC mice; capsaicin inhibits intestinal Cl^−^ secretion and promotes Na^+^ ‐glucose absorption by blocking TRPV4 channels,^54^ thus exerting its beneficial anticolitis effect.

## 7 CONCLUSIONS

Ion channels and transporters play key roles in various physiological processes in the gastrointestinal tract, including Cl^−^ secretion, motility, hormone secretion, etc. They are ideal drug targets for treating several GI diseases, including IBD, colorectal cancer and metabolic syndrome. Ion channels and transporters in IEC have been shown to regulate luminal nutrient uptake in physiological or pathophysiological states, excluding neurological and endocrine roles.

It is proved that K^+^ channels are involved in nutrient absorption, especially in the positive regulation of carbohydrate and protein hydrolysate absorption, by affecting Em after activation. There is still a lack of studies on the involvement of intestinal K^+^ channels or transporters in lipid absorption. Nutrient absorption is often accompanied by Ca^2+^ movement. In turn, increased [Ca^2+^]_i_ caused by activation of Ca^2+^ transporters regulate nutrient absorption from multiple directions, and the following two are considered: firstly, the activity of K^+^ channels or NHE can be controlled to affect the transmembrane potential, and secondly, the movement of nutrient transporters can be directly affected. Therefore, the therapeutic strategy of selecting Ca^2+^ channels or transporters' drugs to adjust nutrient absorption must be reconsidered. A series of ion exchangers (such as NHE3, NBCe1 and PAT‐1) regulate nutrient uptake, especially proteins and lipids hydrolysates, mainly by influencing pH_i_. Taken together, although, in recent years, research on the intestinal ion channels and transporters regulating nutrient absorption to achieve considerable progress, targeting ion channels in disease treatment has promising prospects. In the development of ion channel targeting allows it to be more effective personalized therapy before, we still need to dig deeper into the mechanics.

## AUTHOR CONTRIBUTIONS

**Xianmin Lu:** Writing – original draft (lead). **Chen luo:** Investigation (equal). **Jiangbo Wu:** Resources (supporting). **Ya Deng:** Resources (supporting). **Xingyi Mu:** Resources (supporting). **Ting Zhang:** Resources (supporting). **XiaoXu Yang:** Formal analysis (equal). **Qi Liu:** Resources (supporting). **Zhuo Li:** Resources (supporting). **Siqi Tang:** Resources (supporting). **YanXia Hu:** Resources (supporting). **Qian Du:** Methodology (equal). **JingYu Xu:** Funding acquisition (supporting); project administration (supporting); resources (supporting); supervision (supporting); writing – review and editing (supporting). **Rui Xie:** Funding acquisition (supporting); writing – review and editing (supporting).

## FUNDING INFORMATION

This study was supported by research grants the National Natural Science Foundation of China (No.81660099; No. 82170628; No.81970541; No.31960151; No.32160208; No.81770610) and Collaborative Innovation Center of Chinese Ministry of Education (2020–39). This study was supported by Guizhou Science and Technology Department (Qiankehe platform talents (2021–5647)); This study was supported by Zunyi Science and Technology Bureau (Outstanding Young Talents in Zunyi City (2018–9; 2020–1)); This study was supported by Guizhou Science and Technology Department (Qiankehe foundation‐ZK (2021–major project 004)); This study was supported by Graduate Education and Teaching Innovation Program of Zunyi Medical University (ZYK163).

## CONFLICT OF INTEREST STATEMENT

The authors declare that they have no competing interests.

## CONSENT FOR PUBLICATION

We have obtained consents to publish this paper from all the participants of this study.

## Data Availability

Not applicable.
